# Prediction models for retinopathy of prematurity occurrence based on artificial neural network

**DOI:** 10.1186/s12886-024-03562-y

**Published:** 2024-08-05

**Authors:** Rong Wu, He Chen, Yichen Bai, Yu Zhang, Songfu Feng, Xiaohe Lu

**Affiliations:** 1 Department of Ophthalmology, Zhujiang Hospital, Southern Medical University, No.253 Gongyedadao Middle Road City, Guangzhou, Guangdong 510282 China; 2 Department of Ophthalmology, Peking Union Medical College Hospital, No.5 Summer Palace Road, Beijing, 100000 China

**Keywords:** Retinopathy of prematurity, Artificial neural network, Prediction model, Clinical screening

## Abstract

**Introduction:**

Early prediction and timely treatment are essential for minimizing the risk of visual loss or blindness of retinopathy of prematurity, emphasizing the importance of ROP screening in clinical routine.

**Objective:**

To establish predictive models for ROP occurrence based on the risk factors using artificial neural network.

**Methods:**

A cohort of 591 infants was recruited in this retrospective study. The association between ROP and perinatal factors was analyzed by univariate analysis and multivariable logistic regression. We developed predictive models for ROP screening using back propagation neural network, which was further optimized by applying genetic algorithm method. To assess the predictive performance of the models, the areas under the curve, sensitivity, specificity, negative predictive value, positive predictive value and accuracy were used to show the performances of the prediction models.

**Results:**

ROP of any stage was found in 193 (32.7%) infants. Twelve risk factors of ROP were selected. Based on these factors, predictive models were built using BP neural network and genetic algorithm-back propagation (GA-BP) neural network. The areas under the curve for prediction models were 0.857, and 0.908 in test, respectively.

**Conclusions:**

We developed predictive models for ROP using artificial neural network. GA-BP neural network exhibited superior predictive ability for ROP when dealing with its non-linear clinical data.

## Introduction

Retinopathy of prematurity (ROP) is the leading cause of damaged visual function and blindness in infants, with an incremental incidence owing to improved neonatal intensive care and survival rate of immature infants, but insufficient resources to provide optimal prenatal and perinatal care against ROP [1, 2]. Worldwide an estimated 50 thousand children occur blindness from ROP yearly, majority of whom are from countries in Eastern Europe and Latin America, as well as India and China [2]. In developed countries, the risk of blindness from ROP is < 10%, but can be as high as 40% in developing countries [3]. Early diagnosis and timely treatment are essential for minimizing the risk of visual loss or blindness of ROP, which emphasize the importance of ROP screening in clinical routine.

The screening criteria is based on gestational age and birth weight as well as repeated eye examinations, which differs between countries and regions because of the varying characteristics of the preterm population and neonatal intensive care practices in diverse socioeconomic settings [1]. Repeated ROP screening is associated with invasiveness and pain for the infants, whereas less than 10% of infants screened require treatment under the current criteria [4]. On the other hand, limited medical resources cannot meet the needs with mounting number of infants born prematurely, resulting in 40% of childhood blindness due to insufficient neonatal and ophthalmologic care [5]. Herein lies a pressing need for noninvasive ROP predictive models that could assist identifying high-risk infants and reducing the number of unnecessary screenings safely.

Predictive models of ROP have been developed by incorporating other clinical parameters besides gestational age and birth weight [6–11]. For example, WINROP, one of the most widely validated predictive algorithms, was constructed based on weekly postnatal measurements of serum IGF-1 levels and body weight [7]. Although exhibiting promising predictive performance in previous studies [12–15], its widespread clinical application was limited since WINROP did not include infants with a gestational age at birth of more than 32 weeks. Likewise, the predictive ability of existing models fluctuated with the characteristics of the studied population and disparities in neonatal practices, and thus limited by lack of generalizability and small sample size.

By applying algorithms that mimic the processes of biological neural systems, artificial neural network has proven its reliability and flexibility in several areas, including aiding diagnosis in clinical practice [16–18]. It consists of a set of interconnected “neurons” linked with weighted connections, which can be modified by learning through examples [16]. If there is a discrepancy and an error signal is generated, back propagation (BP) method is applied to alter the weight of the connections between neurons to decrease the overall error of the network [19]. Genetic algorithm (GA) is a directed random search technique that can improve the screening efficiency of BP neural network through optimizing weights and thresholds in dimensionality reduction process [20]. Artificial neural network shows advantages when dealing with nonlinear clinical information [16]. However, it has never been applied for ROP prediction.

The purpose of this study is to establish predictive models for ROP based on the risk factors using artificial neural network, aiming to explore noninvasive and cost-effective methods for accurate prediction identification of ROP.

## Methods

### Data set preparation

All institutional review board approved the protocol in accordance with the principles of the Declaration of Helsinki (revision of Edinburgh, 2000) and all parents or guardians of the recruited infants provided informed written consent before enrollment. Infants with a birth weight less than 2000 g, a gestational age less than 32 weeks, or with risk factors determined by the neonatologists, were included. Exclusion criteria were as following: (1) infants with known or suspected genetic metabolic diseases or chromosome abnormalities; (2) infants who died before all the necessary ROP screenings; (3) infants with other congenital ocular abnormalities; (4) infants with incomplete data. Finally, Six hundred and eighty infants were retrieved from Zhujiang Hospital of Southern Medical University (ZHSMU) and the Second Nanning People Hospital (SNPH), of those, eighty-nine infants were excluded for genetic metabolic diseases, other congenital ocular abnormablities or incomplete data, five hundred and ninety-one infants were enrolled in this study, who were born between September 2014 and September 2018 and received the complete ROP screening examinations.

Clinical data collected for our study involved maternal factors, neonatal factors and neonatal interventions. The maternal factors included maternal age, mode of delivery (cesarean or vaginal), multiple gestations, in vitro fertilization, gestational hypertension, gestational diabetes mellitus, reproductive tract infection during pregnancy, use of antenatal steroids. The neonatal factors included birth weight, gestational age, small for gestational age, sex, intrauterine fetal distress, Apgar scores at five minutes, neonatal asphyxia, respiratory distress syndrome, bronchopulmonary dysplasia, sepsis, necrotizing enterocolitis, intracranial hemorrhage, hypoxic ischemic encephalopathy, patent ductus arteriosus, and hyperbilirubinemia. The neonatal interventions included invasive mechanical ventilation and blood transfusions. All clinical data were collected from electronic medical records The examinations for ROP were conducted by two qualified ophthalmologists. All preterm infants underwent fundus examination according to the Chinese guidelines for ROP screening. The International Classification of Retinopathy of Prematurity 2005 was applied for diagnosis and classification of ROP.

All infants were labeled independently by two clinical ophthalmologists (S.F. and R), with more than 3 years’ experience of ROP care. According to the retinal photographs captured using RetCam III digital fundus camera (Natus, San Carlo, CA, USA), each case was annotated ROP or NO ROP. The κ was 0.81 for annotation of ROP occurrence, suggesting good agreement of the two ophthalmologists in labeling.

### Data processing and development of Prediction models

**Fig. 1:**
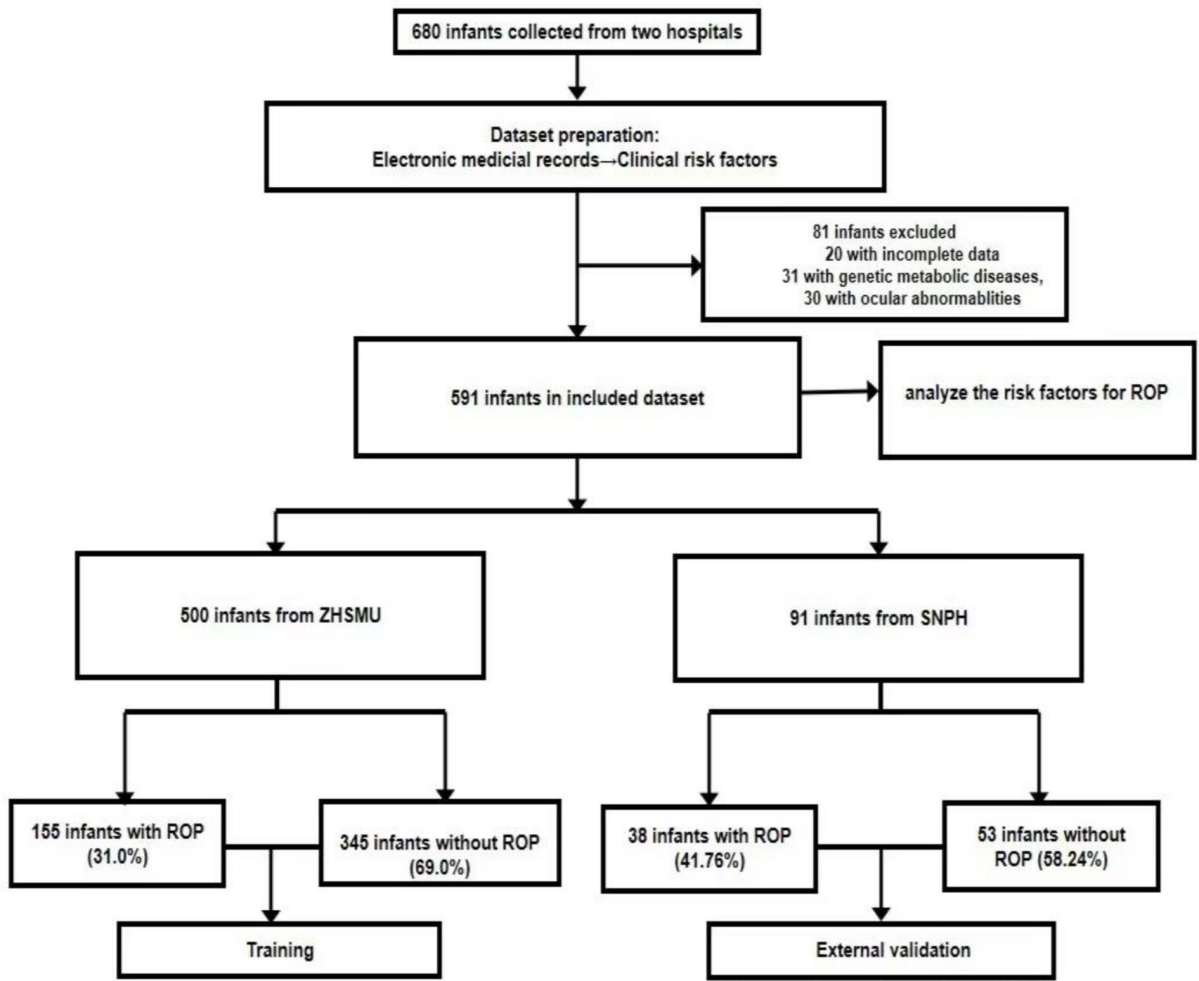
The flowchart of patients’ cohorts

Figure 1 showed the flow chart of the research. First, univariate analysis is performed to analyze the potential predictive factors and compare these factors between non-ROP infants and ROP infants, then multivariate logistic regression analysis is performed to find independent risk factors for ROP occurrence using the factors with statistical difference in univariate analysis.

**Fig. 2:**
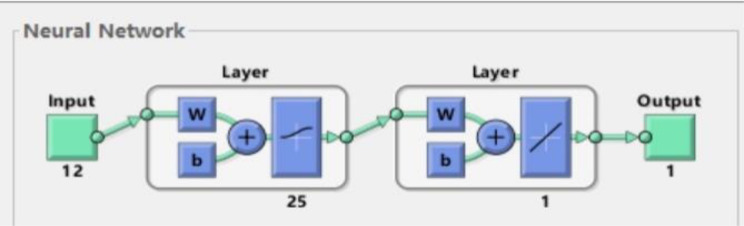
The structure of the BP neural network

The factors with statistical difference in univariate analysis after preprocessing were set as the input variables and ROP or NO ROP (1 = ROP,0 = NO ROP) was set as the output variables. The data mining software package MATLAB (Matrix Laboratory, Math Works Company, USA, R2014a software) was utilized to run artificial neural network models. An artificial neural network model was established with a standard feed-forward BP network structure to mimic the relationship between the risk factors and ROP, including an input layer of 12 nodes, a hidden layer of 25 nodes, and an output layer of one node (Fig. 2). The number of nodes of the hidden layer was decided by repeated trials until the optimal sensitivity and specificity was obtained. The activation function sigmoid was used from the input layer to the hidden layer and purelin was used from the hidden layer to the output layer. Sigmoid transfer functions were applied to the hidden and output layers. Gradient descent was employed to decide the synaptic weights. The initial learning rate was defined as 0.1 and the max training time is 1000.

The genetic algorithm method was applied for optimization of the weights and biases of the BP neural network model. The process of optimizing BP neural network by genetic algorithm mainly includes parameter coding input, selection of initial population and definition of fitness functions and selection, crossover, and mutation operations. The number of individuals was 50. The length of individual coding was 351. When the evolution algebra was 11, the average fitness and the best fitness reached the maximum value; that was, the sum of square error of the test set obtained the least value. To provide a good performance of the prediction model and prevent over-fitting, 500 infants from ZHSMU were used for training, 91 infants from SNPH were used for test.

The activation function sigmoid is.

$$\log sig{\rm{(}}x{\rm{) = 1/(1 + }}{{\rm{e}}^{{\rm{ - x}}}}{\rm{)}}$$

The activation function purelin is

$$\varvec{y}=\varvec{a}\varvec{*}\varvec{x}+\varvec{b}$$

### ROP score prediction model validation

To better demonstrate the prediction accuracy of our model, the ROP Score model is evaluated in test set. ROP Score is a scoring system applied at 6 weeks postnatal age, serves as a prediction model for ROP occurrence and ROP severity, including birth weeks, gestational age, blood transfusion, mechanical ventilation and proportional weight gain at the sixth week postnatal age. The suggested alarm cut-off score is 11 for ROP occurrence and 14.5 for ROP severity [10]. 

### Statistical analysis

Statistical analyses were performed using SPSS 20.0 (IBM Corp., Armonk, NY, USA). Categorical variables were expressed in proportions and analyzed by using Pearson’s $\:{x}^{2}$ test and Fisher’s exact test. As for continuous variables, the normal distribution variables were represented by means and standard deviations, while non-normal ones were expressed as medians and quartile ranges. Continuous variables were analyzed by using independent sample t-test. Multivariate analysis was performed using logistic regression analysis to identify independent risk factors for ROP. Backward step-wise selection was employed with Akaike’s information criterion as the stopping rule. To assess the predictive performance of the models, receiver-operating characteristic (ROC) curves were constructed. In each ROC analysis, the area under the ROC curve (AUC), negative predictive value (NPV), positive predictive value (PPV), accuracy (ACC), sensitivity and specificity were calculated. The identification accuracy referred to the fraction of infants classified correctly. *P* < 0.05 was considered statistically significant.

## Results

In the present study, a total of 1,182 eyes of 591 infants who met the inclusion criteria were enrolled in the analysis. The mean gestational age at birth was 31.0 ± 2.5 weeks, the mean birth weight was 1.5 ± 0.4 kg, and the sex distribution was 374 (63.3%) male and 217 (36.7%) female. ROP of any stage was found in 193 (32.7%) infants, among whom 93 (15.7%) infants had Zone III disease, 70 (12.0%) infants had Zone II disease, and 16 (2.7%) infants had Zone I disease; 66 (11.2%) infants had stage 1, 83 (14.0%) infants had stage 2, 30 (5.1%) infants had stage 3, 14 (2.4%) had aggressive posterior ROP, and none presented ROP at stage 4 or 5.

Univariate analysis was performed to identify the risk factors associated with ROP (Table 1). The result showed that among the maternal factors, cesarean delivery was associated with the presence of ROP (*P* < 0.05), while no difference was found in maternal age, multiple gestations, in vitro fertilization, gestational hypertension, gestational diabetes mellitus, reproductive tract infection during pregnancy, or antenatal steroids use. Besides, the neonatal factors statistically associated with ROP included gestational age, birth weight, Apgar score at five minutes, neonatal asphyxia, bronchopulmonary dysplasia, respiratory distress syndrome, intracranial hemorrhage, hypoxic ischemic encephalopathy, necrotizing enterocolitis, and patent ductus arteriosus (*P* < 0.05). As for the neonatal interventions, both mechanical ventilation and blood transfusions exhibited significant association with the presence of ROP (*P* < 0.05). Multiple logistic regression with the aforementioned risk factors of ROP showed that gestational age, birth weight, bronchopulmonary dysplasia, and hypoxic ischemic encephalopathy were independently associated with the presence of ROP (Table 2).

**Table 1:** Univariate analysis of risk factors for ROP

Variables	NO ROP (n = 398)	ROP (n = 193)	*P*	OR	95%CI
**Maternal factors**					
Maternal age, years	29.90 ± 5.83	30.74 ± 6.09	0.108	NA	NA
Caesarean delivery	195 (49.0)	76 (39.4)	0.028	0.676	0.477–0.959
Twins	97 (24.4)	45 (23.3)	0.778	0.944	0.630–1.414
In vitro fertilization	41 (10.3)	18 (9.3)	0.711	0.896	0.500-1.604
Gestational hypertension	38 (9.5)	24 (12.4)	0.283	1.345	0.782–2.315
Gestational diabetes	55 (13.8)	32 (16.6)	0.374	1.240	0.771–1.992
Reproductive tract infections during pregnancy	34 (8.5)	11 (5.7)	0.222	0.647	0.320–1.307
Antenatal steroids use	84 (21.1)	40 (20.7)	0.915	0.977	0.640–1.492
**Neonatal factors**					
Gestational age, weeks	31.64 ± 2.28	29.53 ± 2.14	<0.001	NA	NA
Birth weight, kg	1.60 ± 0.32	1.29 ± 0.33	<0.001	NA	NA
Small for gestational age	79 (19.8)	38 (19.7)	0.963	0.990	0.643–1.525
Male	249 (62.6)	125 (64.8)	0.602	0.909	0.635–1.301
Intrauterine fetal distress	26 (6.5)	7 (3.6)	0.149	0.538	0.229–1.264
Apgar scores at 5 min	9.16 ± 1.37	8.63 ± 1.71	<0.001	NA	NA
Neonatal asphyxia	85 (21.4)	66 (34.2)	0.001	1.914	1.306–2.804
Respiratory distress syndrome	263 (66.1)	169 (87.6)	<0.001	3.615	2.247–5.814
Bronchopulmonary dysplasia	82 (20.6)	113 (58.5)	<0.001	5.443	3.739–7.924
Sepsis	109 (27.4)	67 (34.7)	0.068	1.410	0.975–2.040
Necrotizing enterocolitis	24 (6.0)	23 (11.9)	0.013	2.108	1.157–3.841
Intraventricular hemorrhage	111 (27.9)	87 (45.1)	<0.001	2.122	1.483–3.307
Hypoxic ischemic encephalopathy	36 (9.0)	38 (19.7)	<0.001	2.465	1.506–4.037
Patent ductus arteriosus	106 (26.6)	69 (35.8)	0.023	1.533	1.060–2.217
Hyperbilirubinemia	284 (71.4)	124 (64.2)	0.080	0.721	0.500–1.040
**Neonatal interventions**					
Mechanical ventilation	297 (74.6)	188 (97.4)	<0.001	12.787	5.114–31.968
Blood transfusion	308 (77.4)	174 (90.2)	<0.001	2.676	1.577–4.540

ROP, retinopathy of prematurity

**Table 2:** Logistic regression analysis of potential risk factors for ROP occurrence

Risk factor	Multivariate analysis		
	OR	95%CI	P Value
Gestational age	0.838	0.729–0.962	0.010
Birth weight	0.261	0.107–0.635	0.003
Bronchopulmonary dysplasia	2.031	1.306–3.157	0.002
Hypoxic ischemic encephalopathy	3.618	1.357–9.521	0.009

**Fig. 3:**
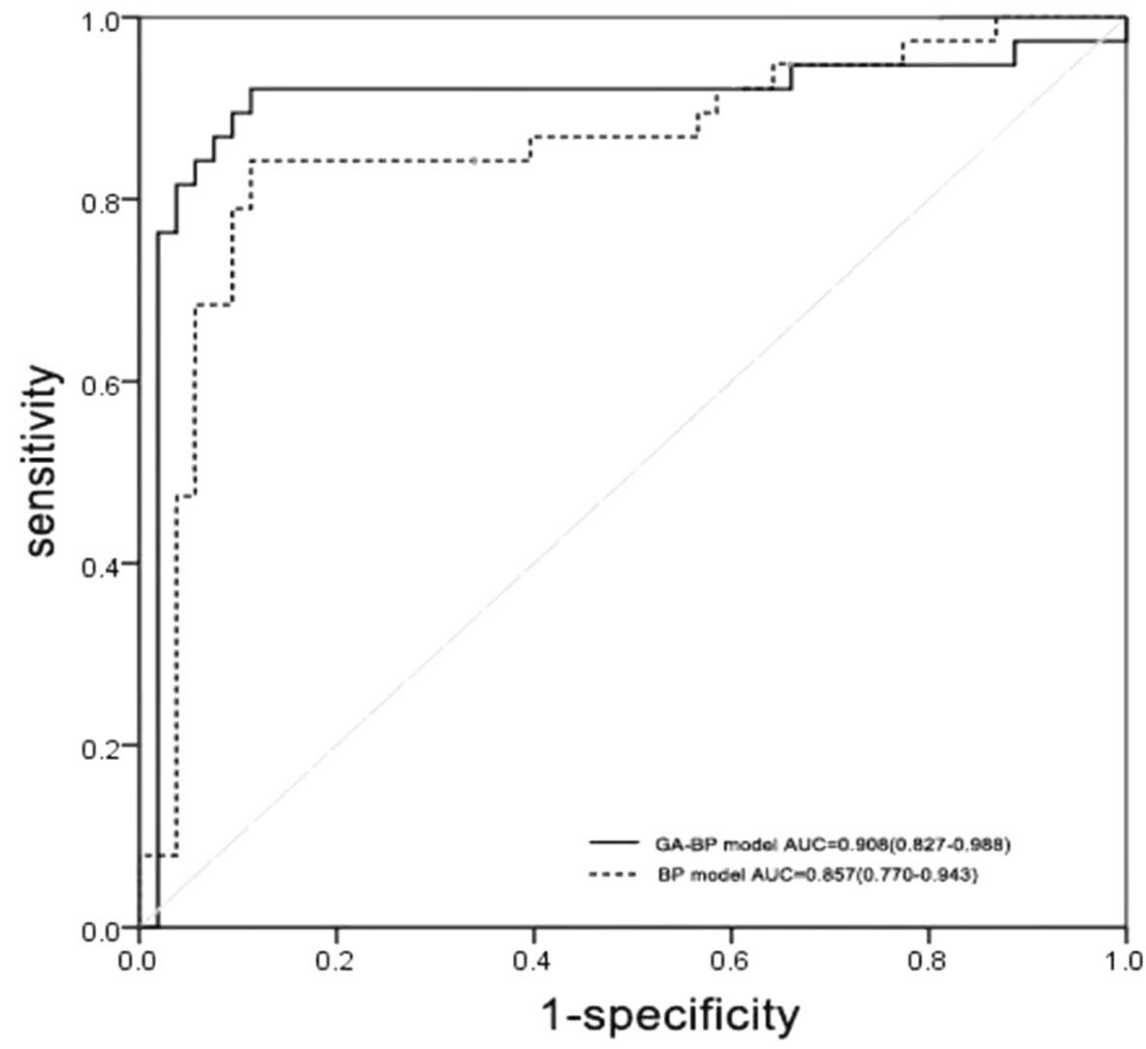
Receiver operating characteristic curves of predictive models

**Fig. 4:**
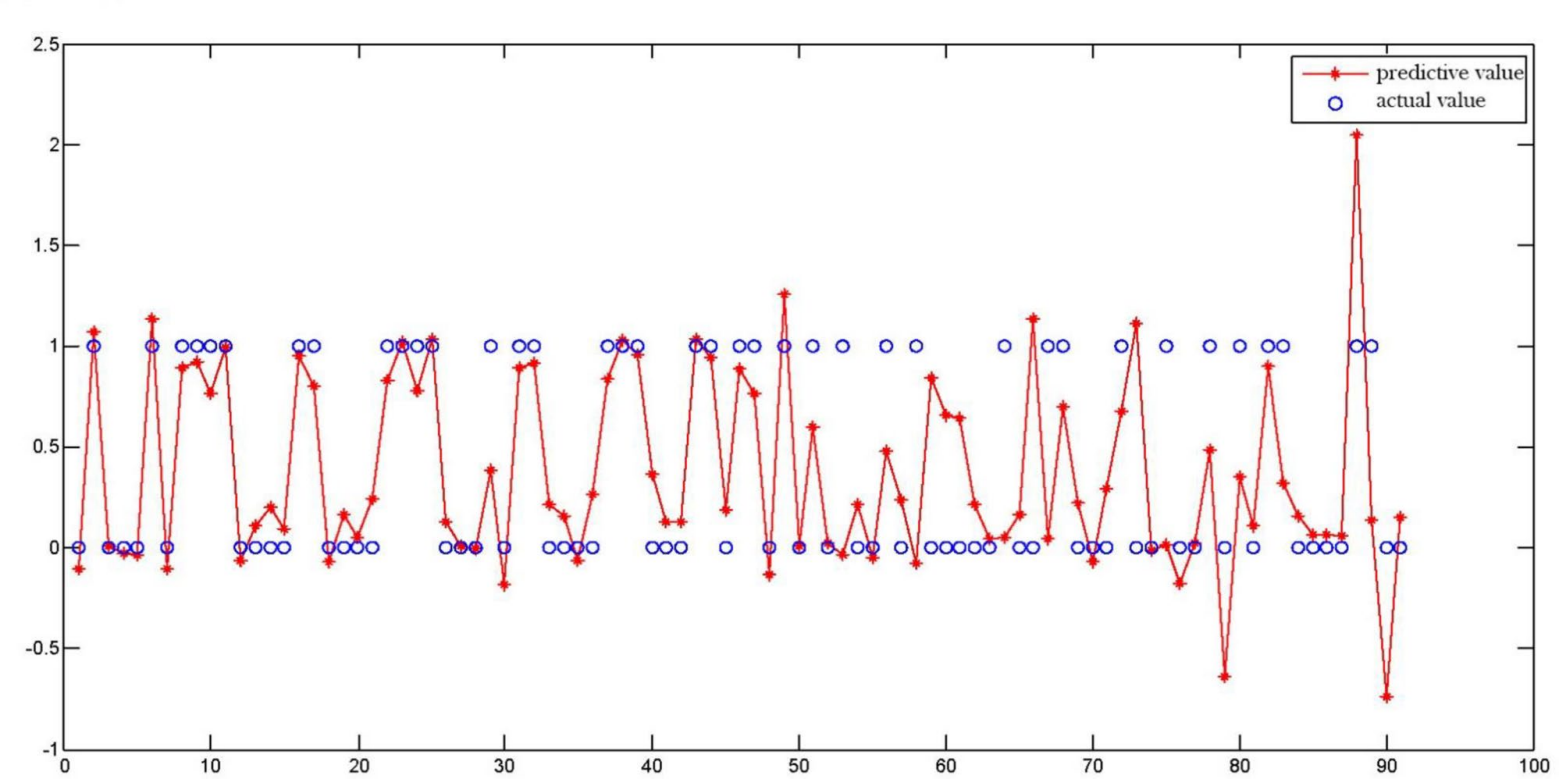
The contrast of actual values and predictive values for BP models

**Fig. 5:**
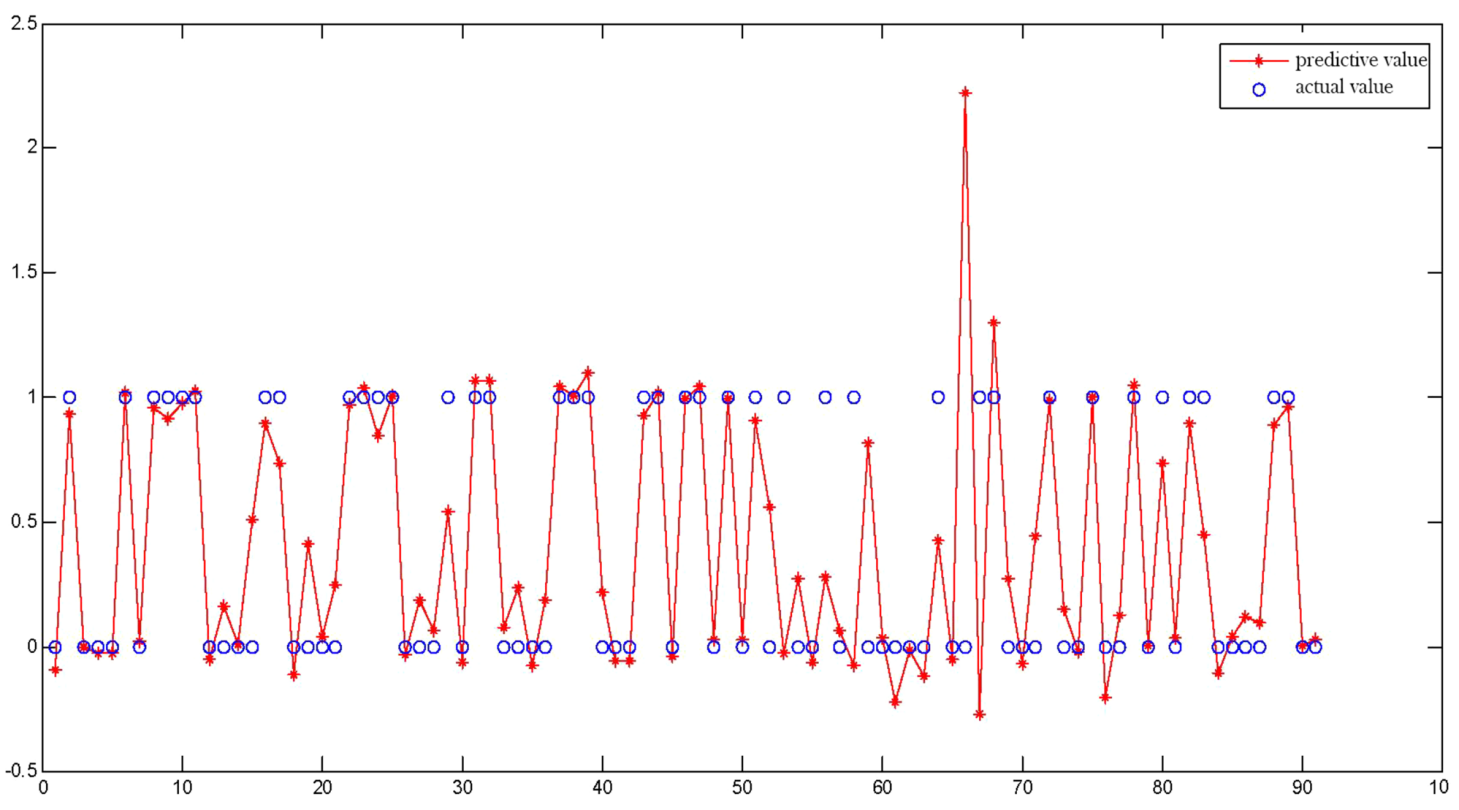
The contrast of the actual values and predictive values for GA-BP models

After applying prediction models in external validation cohorts, the ROC curves for BP and genetic algorithm-back propagation (GA-BP) models were presented in Fig. 3. After applying the threshold of 0.307 for BP prediction model and 0.276 for GA-BP model, these two models get the best predictive performances. The predictive performances of these models for ROP were displayed in Table 3. The GA-BP model demonstrated the best performance for detecting ROP with an AUC of 0.908 (95% CI 0.827–0.988). In comparison, the AUC value was 0.557(95% CI 0.434–0.579) for the ROP Score model, and 0.857 (95% CI 0.770–0.943) for the BP model. The contrast of the actual values and predictive values for BP and GA-BP models is in Figs. 4 and 5.

**Table 3:** Performance of the predictive models for ROP screening

	BP model	GA-BP model	ROP Score
AUC (95%CI)	0.857(0770-0.943)	0.908(0.827–0.998)	0.557(0.434–0.679)
Specificity	88.7%	88.7%	30.2%
Sensitivity	84.2%	92.1%	84.2%
NPV	88.7%	94.0%	72.73%
PPV	84.2%	85.4%	46.4%
ACC	86.8%	90.1%	52.7%

ROP, retinopathy of prematurity; BP, back propagation; GA-BP, genetic algorithm-back propagation; AUC, the area under the receiver operating characteristic curve; NPV, negative predictive value; PPV, positive predictive value. CI, confidence interval;

## Discussion

ROP is a neurovascular disease of the retina affecting preterm infants, which is a predominant etiology of childhood blindness and vision impairment globally. In 2010, an estimated 184,700 infants of 14.9 million premature infants developed any stage of ROP worldwide, 20,000 of whom suffered blindness or severe visual impairment from ROP [21] Timely screening of ROP is crucial, whereas barriers exist to its effective implementation, including scarce health resources in low- and middle-income regions, and unnecessary examination under varying screening criteria across the world [1]. Currently, noninvasive prediction of ROP has been highlighted, aiming to safely ease the screening burden without missing severe infants, especially for regions where the number of ophthalmologists is limited and the conventional ROP screening is not readily accessible.

Some effective and safe screening criterion has been proposed to optime ROP screening [22, 23]. A recent study reported an individual predictive model based on gestational age, birth weight, and sex for infants with a gestational age of 24 to 30 weeks, enabling early risk estimation for receiving ROP treatment [22]. The predictive ability of this novel model was confirmed by test with excellent AUCs varying from 0.84 to 0.94. Furthermore, the input variables were simple, facilitating the general use of the model. However, infants born at gestational age < 24 weeks or > 31 weeks could not be included. In China, more mature and larger infants are also at risk of ROP [24]. Therefore, the inclusion criteria in our study were broader than the screening guidelines recommended in the USA (gestational age < 30 weeks, birth weight < 1500 g) and UK (gestational age < 32 weeks, birth weight < 1500 g) [25, 26]. Totally, 591 infants with mean gestational age and birth weight of 31.0 ± 2.5 weeks and 1.5 ± 0.4 kg, respectively, were included.

The pathogenesis of ROP is still not fully unveiled, which appears to be multi-factorial and is associated with a number of epigenetic modifications by perinatal factors [27]. In the present study, 12 risk factors of ROP were selected, including gestational age, birth weight, caesarean delivery, Apgar score at five minutes, bronchopulmonary dysplasia, respiratory distress syndrome, intracranial hemorrhage, hypoxic ischemic encephalopathy, mechanical ventilation, blood transfusions, necrotizing enterocolitis, and patent ductus arteriosus. Previous studies have examined the interrelationships of the various independent risk factors in the presence of ROP. Giapros et al. demonstrated that ROP was independently related to both low gestational age and the presence of chronic lung disease, which were interrelated in the development of ROP. The combined risk effect on ROP was found to be larger than the sum of the individual risk effects, revealing that there was a positive interaction on an additive scale [27]. In the study of Chen et al and Giapros, sepsis, oxygen exposure, and low gestational age, were found to be independently associated with a significantly increased risk of ROP with significant interrelationships [28, 29]. Slidsborg et al. described that mechanical ventilation and blood transfusion were statistically independent risk factors for ROP development [30], which were proved by many studies [31–33]. These findings might imply that artificial neural network, which simulates the function and structure of biological neural network to establish non-linear mathematical models could perform better when constructing predictive models of ROP with various pathogenic factors and complicated interrelationships among them [16].

Nowadays, BP neural network algorithm, a multi-layer feed forward network, has been widely used in the field of medicine, especially to assist diagnosis and clinical decision-making, with its strong fault tolerance, adaptiveness, nonlinear comprehensive reasoning ability, and the powerful ability to solve co-linearity and interactions between variables [19]. Yao et al. proposed a BP neural network for predicting diabetic retinopathy based on biochemical and metabolic parameters, which achieved an AUC of 0.84 with a sensitivity of 0.73 and a specificity of 0.83 [34]. Nevertheless, further improvement is required concerning its limited study cohort and unsatisfactory sensitivity before its application in screening practice. In the present study, based on the risk factors extraction, predictive models were established using BP neural networks, and GA-BP neural networks, respectively, and displayed encouraging performance on detection of ROP with reasonable AUCs. In comparison to the logistic regression model, the BP neural network model exhibited superior performance for ROP prediction, which was further improved by applying GA-BP neural network with higher sensitivity, accuracy and AUC. We also validated and compared the prediction performances of ROP score prediction model with our prediction models, the AUC, sensitivity, specificity and accurancy of our prediction models and ROP Score were comparable in predicting ROP.

A potential criticism of our study is the small sample size, which may bring the doubt of the optimal cut-off value and predictive ability of the models owing to insufficient machine learning. Artificial neural networks would be better trained using large-scale datasets [16]. The proposed models need to be further validated in larger cohorts. Another limitation of our study is that ROP risk-stratification for optimization of timing and frequency of the screening examinations and accurate treatment cannot be realized by our predictive models, which might be explored in our future study to better represent the current clinical scenario and achieve the goal of precision medicine. There are several limitations for artificial neural network. First, the artificial neural network model is like a “black box” that cannot determine causality, it is impossible to give a probability of a predicted outcome, provides almost no medical explanation for the variables, and is difficult to go into depth and professional context Knowledge combination. Second, artificial neural network uses the method of iterative update to determine the weight, so an initial value is required, generally at the beginning, the initial values are given randomly, which tends to cause the non-reproducibility of the network [35].

In conclusion, we developed predictive models for noninvasive ROP detection using BP neural network, and GA-BP neural network, respectively, with the hope to safely spare unnecessary screening. GA-BP neural network exhibited superior predictive ability for ROP when dealing with its non-linear clinical data.

## Data Availability

The datasets are not publicly available due to the reason that the data is also part of an ongoing study but are available from the corresponding author on reasonable request.
